# Supplementary material for: Abdominal massage for neurogenic bowel dysfunction in people with multiple sclerosis (AMBER — Abdominal Massage for Bowel Dysfunction Effectiveness Research): study protocol for a randomised controlled trial
Source: Trials. 2017 Mar 29;18:150. doi: 10.1186/s13063-017-1890-y (PMC5372315; doi:10.1186/s13063-017-1890-y)
Supplement: Additional file 1: — SPIRIT 2013 checklist: recommended items to address in a clinical trial protocol and related documents. (DOC 124 kb) [file 13063_2017_1890_MOESM1_ESM.doc]

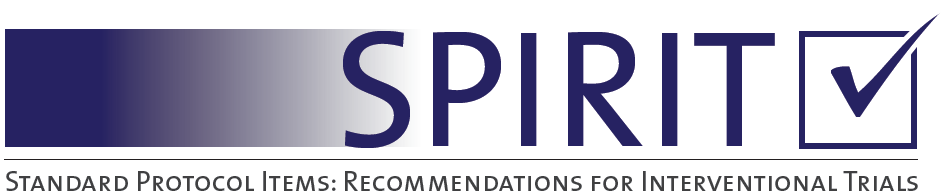


SPIRIT 2013 Checklist: Recommended items to address in a clinical trial protocol and related documents*

| Section/item | ItemNo | Description |
| --- | --- | --- |
| **Administrative information** | | |
| Title | 1✔ | Descriptive title identifying the study design, population, interventions, and, if applicable, trial acronym  Abdominal massage for neurogenic bowel dysfunction in people with multiple sclerosis  (AMBER - Abdominal Massage for Bowel Dysfunction Effectiveness Research) |
| Trial registration | 2a✔ | Trial identifier and registry name. If not yet registered, name of intended registry  http://www.controlled  trials.com/ISISRCTN85007023 on the 26th June |
|  | 2b | All items from the World Health Organization Trial Registration Data Set N/A |
| Protocol version | 3✔ | Date and version identifier  V5 02.02.17 |
| Funding | 4✔ | Sources and types of financial, material, and other support  The trial was funded by the United Kingdom National Health Service through the  National Institute for Health Research Health Technology Assessment programme,  open call Project number HTA 12/127 |
| Roles and responsibilities | 5a✔ | Names, affiliations, and roles of protocol contributors  Doreen McClurg, PhD – Chief Investigator  Kirsteen Goodman, PhD – Trial Manager  Suzanne Hagen, PhD – co-applicant and trials expert  Fiona Harris, PhD – QoL lead  Shaun Treweek, PhD- expert in trial recruitment/retention  Anton Emmanuel, MBBS Medicine – Consultant ano-rectal dysfunction, Local PI  Christine Norton, PhD – Bowel dysfunction expert  John Norrie, MSc statistics – statistical and trials expert  Peter Donnan, PhD Medical Statistics – CTU manager  Maureen Coggrave, PhD – Neurogenic bowel expert  Sarkis Manoukian, PhD – Health economics |
|  | 5b✔ | Name and contact information for the trial sponsor – Yasmin Glover, RIE, Glasgow Caledonian University, Glasgow G4 0BA |
|  | 5c✔ | Role of study sponsor and funders, if any, in study design; collection, management, analysis, and interpretation of data; writing of the report; and the decision to submit the report for publication, including whether they will have ultimate authority over any of these activities  The funders reviewed the application and suggested amendments to the protocol etc which were undertaken. The funders have reviewed the submission and approved it. |
|  | 5d✔ | Composition, roles, and responsibilities of the coordinating centre, steering committee, endpoint adjudication committee, data management team, and other individuals or groups overseeing the trial, if applicable (see Item 21a for data monitoring committee)  The CTU is Tayside Clinical Trials Unit ,and is providing trial support, data management an statistical expertise  The role of the TSC/SSC is to provide overall supervision for a trial on behalf of the Trial Sponsor and Trial Funder and to ensure that the trial is conducted to the rigorous standards set out in the Department of Health’s Research Governance Framework for Health and Social Care and the Guidelines for Good Clinical Practice. Prof John Saxton (Chair), Ms Marian Emly, Prof Richard Morris, Mr Ashley Pollock, Ms Cassie Sincock, (PPI) and Ms Gail Hunter (PPI) + Co-applicants  The DMC is completely independent and comprises of 3 experts in the field of clinically, expert trial statistician, and will monitor data and make recommendations to the TSC if there are any ethical or safety issues. Dr Christ Sutton (Chair), Ms Diane Stark, Prof Lorna Paul |
| Introduction |  |  |
| Background and rationale | 6a✔ | Description of research question and justification for undertaking the trial, including summary of relevant studies (published and unpublished) examining benefits and harms for each intervention  Explained in Paper |
|  | 6b✔ | Explanation for choice of comparators Explained in Paper |
| Objectives | 7✔ | Specific objectives or hypotheses Explained in paper |
| Trial design | 8✔ | Description of trial design including type of trial (eg, parallel group, crossover, factorial, single group), allocation ratio, and framework (eg, superiority, equivalence, noninferiority, exploratory) Explained in Paper |
| Methods: Participants, interventions, and outcomes | | |
| Study setting | 9✔ | Description of study settings (eg, community clinic, academic hospital) and list of countries where data will be collected. Reference to where list of study sites can be obtained Explained in Paper |
| Eligibility criteria | 10✔ | Inclusion and exclusion criteria for participants. If applicable, eligibility criteria for study centres and individuals who will perform the interventions (eg, surgeons, psychotherapists) Explained in Paper |
|  | 11a✔ | Interventions for each group with sufficient detail to allow replication, including how and when they will be administered Explained in Paper |
|  | 11b✔ | Criteria for discontinuing or modifying allocated interventions for a given trial participant (eg, drug dose change in response to harms, participant request, or improving/worsening disease) Explained in Paper |
|  | 11c✔ | Strategies to improve adherence to intervention protocols, and any procedures for monitoring adherence (eg, drug tablet return, laboratory tests) Bowel diaries also record adherence to undertaking massage |
|  | 11d✔ | Relevant concomitant care and interventions that are permitted or prohibited during the trial Explained in Paper |
| Outcomes | 12  ✔ | Primary, secondary, and other outcomes, including the specific measurement variable (eg, systolic blood pressure), analysis metric (eg, change from baseline, final value, time to event), method of aggregation (eg, median, proportion), and time point for each outcome. Explanation of the clinical relevance of chosen efficacy and harm outcomes is strongly recommended Explained in Paper |
| Participant timeline | 13✔ | Time schedule of enrolment, interventions (including any run-ins and washouts), assessments, and visits for participants. A schematic diagram is highly recommended (see Figure) Explained in Paper |
| Sample size | 14✔ | Estimated number of participants needed to achieve study objectives and how it was determined, including clinical and statistical assumptions supporting any sample size calculations Explained in Paper |
| Recruitment | 15✔ | Strategies for achieving adequate participant enrolment to reach target sample size Explained in Paper |
| **A** | | |
| Allocation: |  |  |
| Sequence generation | 16a✔ | Method of generating the allocation sequence (eg, computer-generated random numbers), and list of any factors for stratification. To reduce predictability of a random sequence, details of any planned restriction (eg, blocking) should be provided in a separate document that is unavailable to those who enrol participants or assign interventions Explained in Paper |
| Allocation concealment mechanism | 16b✔ | Mechanism of implementing the allocation sequence (eg, central telephone; sequentially numbered, opaque, sealed envelopes), describing any steps to conceal the sequence until interventions are assigned Explained in Paper |
| Implementation | 16c✔ | Who will generate the allocation sequence, who will enrol participants, and who will assign participants to interventions Explained in Paper |
| Blinding (masking) | 17a✔ | Who will be blinded after assignment to interventions (eg, trial participants, care providers, outcome assessors, data analysts), and how Explained in Paper |
|  | 17b✔ | If blinded, circumstances under which unblinding is permissible, and procedure for revealing a participant’s allocated intervention during the trial N/A |
| **Methods: Data collection, management, and analysis** | | |
| Data collection methods | 18a✔ | Plans for assessment and collection of outcome, baseline, and other trial data, including any related processes to promote data quality (eg, duplicate measurements, training of assessors) and a description of study instruments (eg, questionnaires, laboratory tests) along with their reliability and validity, if known. Reference to where data collection forms can be found, if not in the protocol Explained in Paper |
|  | 18b | Plans to promote participant retention and complete follow-up, including list of any outcome data to be collected for participants who discontinue or deviate from intervention protocols Explained in Paper |
| Data management | 19✔ | Plans for data entry, coding, security, and storage, including any related processes to promote data quality (eg, double data entry; range checks for data values). Reference to where details of data management procedures can be found, if not in the protocol Explained in Paper |
| Statistical methods | 20a✔ | Statistical methods for analysing primary and secondary outcomes. Reference to where other details of the statistical analysis plan can be found, if not in the protocol Explained in paper |
|  | 20b✔ | Methods for any additional analyses (eg, subgroup and adjusted analyses) Explained in Paper |
|  | 20c✔ | Definition of analysis population relating to protocol non-adherence (eg, as randomised analysis), and any statistical methods to handle missing data (eg, multiple imputation) Explained in Paper |
| **Methods: Monitoring** | | |
| Data monitoring | 21a✔ | Composition of data monitoring committee (DMC); summary of its role and reporting structure; statement of whether it is independent from the sponsor and competing interests; and reference to where further details about its charter can be found, if not in the protocol. Alternatively, an explanation of why a DMC is not needed See above and Explained in Paper |
|  | 21b✔ | Description of any interim analyses and stopping guidelines, including who will have access to these interim results and make the final decision to terminate the trial Explained in Paper |
| Harms | 22✔ | Plans for collecting, assessing, reporting, and managing solicited and spontaneously reported adverse events and other unintended effects of trial interventions or trial conduct Explained in Paper |
| Auditing | 23 | Frequency and procedures for auditing trial conduct, if any, and whether the process will be independent from investigators and the sponsor – DMC and TSC will meet every 6 months |
| Ethics and dissemination | | |
| Research ethics approval | 24✔ | Plans for seeking research ethics committee/institutional review board (REC/IRB) approval West of Scotland Research Ethics Committee 4, obtained on the 11^th^ June 2014 (14/WS/0111) |
| Protocol amendments | 25✔ | Plans for communicating important protocol modifications (eg, changes to eligibility criteria, outcomes, analyses) to relevant parties (eg, investigators, REC/IRBs, trial participants, trial registries, journals, regulators) Funders, sponsors and R&D will be notified routinely and appropriate approvals gained |
| Consent or assent | 26a✔ | Who will obtain informed consent or assent from potential trial participants or authorised surrogates, and how (see Item 32) Explained in Paper |
|  | 26b✔ | Additional consent provisions for collection and use of participant data and biological specimens in ancillary studies, if applicable N/A |
| Confidentiality | 27✔ | How personal information about potential and enrolled participants will be collected, shared, and maintained in order to protect confidentiality before, during, and after the trial As in paper |
| Declaration of interests | 28✔ | Financial and other competing interests for principal investigators for the overall trial and each study site None declared |
| Access to data | 29✔ | Statement of who will have access to the final trial dataset, and disclosure of contractual agreements that limit such access for investigators As in paper |
| Ancillary and post-trial care | 30✔ | Provisions, if any, for ancillary and post-trial care, and for compensation to those who suffer harm from trial participation N/A |
| Dissemination policy | 31a✔ | Plans for investigators and sponsor to communicate trial results to participants, healthcare professionals, the public, and other relevant groups (eg, via publication, reporting in results databases, or other data sharing arrangements), including any publication restrictions Explained in Paper. We have since developed a publication and dissemination policy and have been in touch with relevant patient and clinical interest groups |
|  | 31b✔ | Authorship eligibility guidelines and any intended use of professional writers Explained in Paper |
|  | 31c | Plans, if any, for granting public access to the full protocol, participant-level dataset, and statistical code N/A |
| Appendices |  |  |
| Informed consent materials | 32✔ | Model consent form and other related documentation given to participants and authorised surrogates Attached as an appendix |
| Biological specimens | 33 | Plans for collection, laboratory evaluation, and storage of biological specimens for genetic or molecular analysis in the current trial and for future use in ancillary studies, if applicable N/A |

*It is strongly recommended that this checklist be read in conjunction with the SPIRIT 2013 Explanation & Elaboration for important clarification on the items. Amendments to the protocol should be tracked and dated. The SPIRIT checklist is copyrighted by the SPIRIT Group under the Creative Commons “[Attribution-NonCommercial-NoDerivs 3.0 Unported](http://www.creativecommons.org/licenses/by-nc-nd/3.0/)” license.
